# The neural coding of creative idea generation across adolescence and early adulthood

**DOI:** 10.3389/fnhum.2013.00905

**Published:** 2013-12-30

**Authors:** Sietske W. Kleibeuker, P. Cédric M. P. Koolschijn, Dietsje D. Jolles, Carsten K. W. De Dreu, Eveline A. Crone

**Affiliations:** ^1^Brain and Development Lab, Department of Psychology, Leiden UniversityLeiden, Netherlands; ^2^Leiden Institute for Brain and CognitionLeiden, Netherlands; ^3^Brain and Cognition, University of AmsterdamAmsterdam, Netherlands; ^4^Department of Psychology, University of AmsterdamAmsterdam, Netherlands

**Keywords:** creative cognition, divergent thinking, fMRI, adolescence

## Abstract

Creativity is considered key to human prosperity, yet the neurocognitive principles underlying creative performance, and their development, are still poorly understood. To fill this void, we examined the neural correlates of divergent thinking in adults (25–30 years) and adolescents (15–17 years). Participants generated alternative uses (AU) or ordinary characteristics (OC) for common objects while brain activity was assessed using fMRI. Adults outperformed adolescents on the number of solutions for AU and OC trials. Contrasting neural activity for AU with OC trials revealed increased recruitment of left angular gyrus, left supramarginal gyrus, and bilateral middle temporal gyrus in both adults and adolescents. When only trials with *multiple* AU were included in the analysis, participants showed additional left inferior frontal gyrus (IFG)/middle frontal gyrus (MFG) activation for AU compared to OC trials. Correspondingly, individual difference analyses showed a positive correlation between activations for AU relative to OC trials in left IFG/MFG and divergent thinking performance and activations were more pronounced in adults than in adolescents. Taken together, the results of this study demonstrated that creative idea generation involves recruitment of mainly left lateralized parietal and temporal brain regions. Generating multiple creative ideas, a hallmark of divergent thinking, shows additional lateral PFC activation that is not yet optimized in adolescence.

## Introduction

Creative performance—generating ideas, solutions, and insights that are both novel and useful (Sternberg and Lubart, 1996)—is key to human survival and prosperity. For example, creativity predicts success in conflict situations (De Dreu and Nijstad, 2008), academic success (Furnham and Bachtiar, 2008) and serves important adaptive purposes (Runco, 2004). However, despite its importance for an extensive range of domains of life, the neurocognitive foundations of creative performance are still poorly understood. Furthermore, we know exceedingly little about the developmental trajectories in creative performance. Accordingly, the present study examined neural correlates of divergent thinking performance—a critical ingredient of creative performance (Baas et al., 2008) with significant predictive value for creative success (e.g., Kim, 2008)—in adolescents and adults.

Creativity research distinguishes a variety of approaches, associated with different views concerning the components underlying creative success. Creativity is sometimes considered to be an attribute of a few brilliant minds, and a result of deviant brain functioning. In contrast, the *creative cognition* approach emphasizes that creative capacity is inherent to normative human cognitive functioning and that relevant processes are open to investigation. Our exceptional flexible use of language, our ability to create and use new mental categories to organize our experiences, and our ability to mentally manipulate objects are only some examples of mundane forms of creativity that support the creative cognition approach (Ward et al., 1999). These creative outcomes are a function of a variety of cognitive and motivational processes.

De Dreu and colleagues distinguished two pathways of processes that breed creative outcomes: *flexible processing* and *perseverance*, which are summarized in the Dual Pathway to Creativity Model (DPCM) (Baas et al., 2008; Nijstad et al., 2010). Here, creative performance including the generation of original ideas and creative insights are related to flexible, divergent thinking on the one hand and persistent, bottom-up processing on the other. The *flexibility pathway* involves the generation of novel ideas and creative insights through the use of extensive cognitive categories, flexible switching between categories and strategies, and the use of distant (rather than close) associations (e.g., Mednick, 1962; Koestler, 1964; Amabile, 1983; Eysenck, 1993; Simonton, 1999). Indeed, creativity is often associated with deviating from traditional procedures or “breaking set,” and with overcoming cognitive biases or “functional fixedness” (e.g., Duncker, 1945; Wertheimer, 1945; Smith and Blankenship, 1991). The *persistence pathway* encompasses the processes that lead to creative ideas, insights and problem solutions through systematic and effortful exploration of possibilities within only a few categories or perspectives (Newell and Simon, 1972; Finke, 1996; Simonton, 1997; Boden, 1998; Dietrich, 2004). For example, De Dreu et al. (2012; also see Oberauer et al., 2008) showed that working memory capacity predicted creative performance when time-on-task was long rather than short, because working memory capacity enabled individuals to persist and persevere in generating ideas and creative insights.

Although DPCM identifies creative processes at a cognitive level, the neural processes and developmental trajectories are still poorly understood. Here we explore neural correlates of performing a widely used creativity task- the Alternative Uses Test (AUT) (Guilford, 1967). The AUT asks people to generate as many as possible alternative uses (AU) for a common object (e.g., a brick; with an alternative usage being, e.g., making music). Ideas generated during such a divergent thinking task are commonly coded for originality (the less frequent the idea being mentioned, the more original it is), flexibility (the more uses from different semantic categories, the more flexible someone is), and fluency (the more ideas, the more fluent). Neuroimaging research on creative cognition research in general and divergent thinking in particular has revealed varied results. The most consistent finding across verbal divergent thinking paradigms is the involvement of (left) temporo-parietal regions, including angular gyrus (AG) and supramarginal gyrus (SMG) (Arden et al., 2010; Dietrich and Kanso, 2010). Activity changes of these brain areas have for example been observed during tasks that require linking incoherent sentences (Sieborger et al., 2007) or words (Starchenko et al., 2003; Bechtevera et al., 2004) into a coherent story, creating metaphors and analogies (Hansen et al., 2008), or thinking of AU for common objects (Fink et al., 2009, 2010; Abraham et al., 2012). Fink et al. (2009, 2010) compared activity during the generation of AU with activity during the retrieval of ordinary characteristics (OC), which is thought to be more related to intelligence in general. Results showed increased activity in left AG and SMG, and decreased activity in right AG for generating AU relative to retrieving OC. Another finding that is relatively consistent across various divergent thinking studies concerns the involvement of prefrontal cortex (PFC) (e.g., Carlsson et al., 2000; Folley and Park, 2005; Howard-Jones et al., 2005; Chavez-Eakle et al., 2007; Mashal et al., 2007; Abraham et al., 2012), a brain region that is generally associated with cognitive control functioning and coordinating lower level (associative) brain regions (e.g., Miller and Cohen, 2001). Notably, a substantial part of the studies that put forward the significance of prefrontal recruitment revealed positive relations between PFC activations and creative performances (e.g., Carlsson et al., 2000; Chavez et al., 2004; Chavez-Eakle et al., 2007; Gibson et al., 2009). In all, these results indicate that temporo-parietal regions are involved in divergent thinking processes in general whereas the ability to recruit PFC successfully might be discriminative concerning creative capacities.

Interestingly, in a prior behavioral study we showed that adults were more successful than adolescents in generating original ideas, although there were no age differences in fluency and flexibility (Kleibeuker et al., 2013). One hypothesis is that these age differences are associated with immature cognitive control processes and related prefrontal brain functioning. PFC regions are upon the latest to mature; structural (gray and white matter) and functional changes have been observed throughout adolescence and into adulthood (Shaw et al., 2008; Giedd and Rapoport, 2010; Luna et al., 2010). Age related changes of PFC activations have been observed for several cognitive functions including working memory, interference control and task-switching (for a review, see Bunge and Wright, 2007). Therefore, we differentiated in our analyses between adolescents and adults in order to understand how possible developmental differences in divergent thinking are associated with neural activity in the PFC and, possibly, temporo-parietal regions including left AG and SMG. Specifically, we conducted an fMRI study in which adults and adolescents were asked to provide AU or OC for common objects (Fink et al., 2009, 2010). To reveal brain regions involved in creative cognition, activity for AU generation was contrasted with activity for OC retrieval. According to previous studies, we expected to find activation in left AG and left SMG for alternative generation relative to ordinary characteristic retrieval. To better understand the processes underlying the *divergent* aspect of creative idea generation, we investigated activation patterns for the generation of *multiple* creative ideas, which specifically requires switching between solutions. Based on prior research results, we anticipated lateral PFC activations to be positively associated with divergent thinking performance in both adults and adolescents and to be larger in adults than in adolescents.

## Methods

### Participants

Forty-five right-handed participants with no self-reported history of neurological or psychiatric disorders participated in the present study, divided across two age groups: 25 adolescents (15–17-year-olds) and 20 adults (25–30-year-olds). Analyses involved 43 participants; 24 adolescents (*M*_age_ = 16.89 years, *SD* = 0.63, 12 male), and 19 adults (*M*_age_ = 26.83 years, *SD* = 1.37, 9 male). One adolescent was excluded from the analysis due to technical failures, and one adult was excluded because of excessive head motion (>1.75 mm). Gender distributions did not differ between age groups [χ^2^_(1)_ = 0.03, *p* = 0.86].

Participants were recruited from local schools and through local advertisements. All participants provided informed consent. In case of minors, consent was also obtained from primary caregivers. Participation was compensated with either money or course credits. All procedures were approved by the Internal Review Board of Leiden University Medical Center (LUMC).

To obtain an estimate of intelligence we included two subscales of the Wechsler Adult Intelligence Scale (Digit Span and Similarities; Wechsler, 1991, 1997; see Soveri et al., 2011). The scaled intelligence scores did not differ between age groups [Adolescents: *M* = 24.54, *SD* = 2.32; Adults: *M* = 25.89, *SD* = 4.16; *t*_(41)_ = 1.27, *p* = 0.22, corrected for unequal variances] or gender [Males: *M* = 25.48, *SD* = 2.93; females: *M* = 24.82, *SD* = 3.65; *t*_(41)_ = 0.65, *p* = 0.29].

### Tasks

#### Scanner task

**Figure 1:**
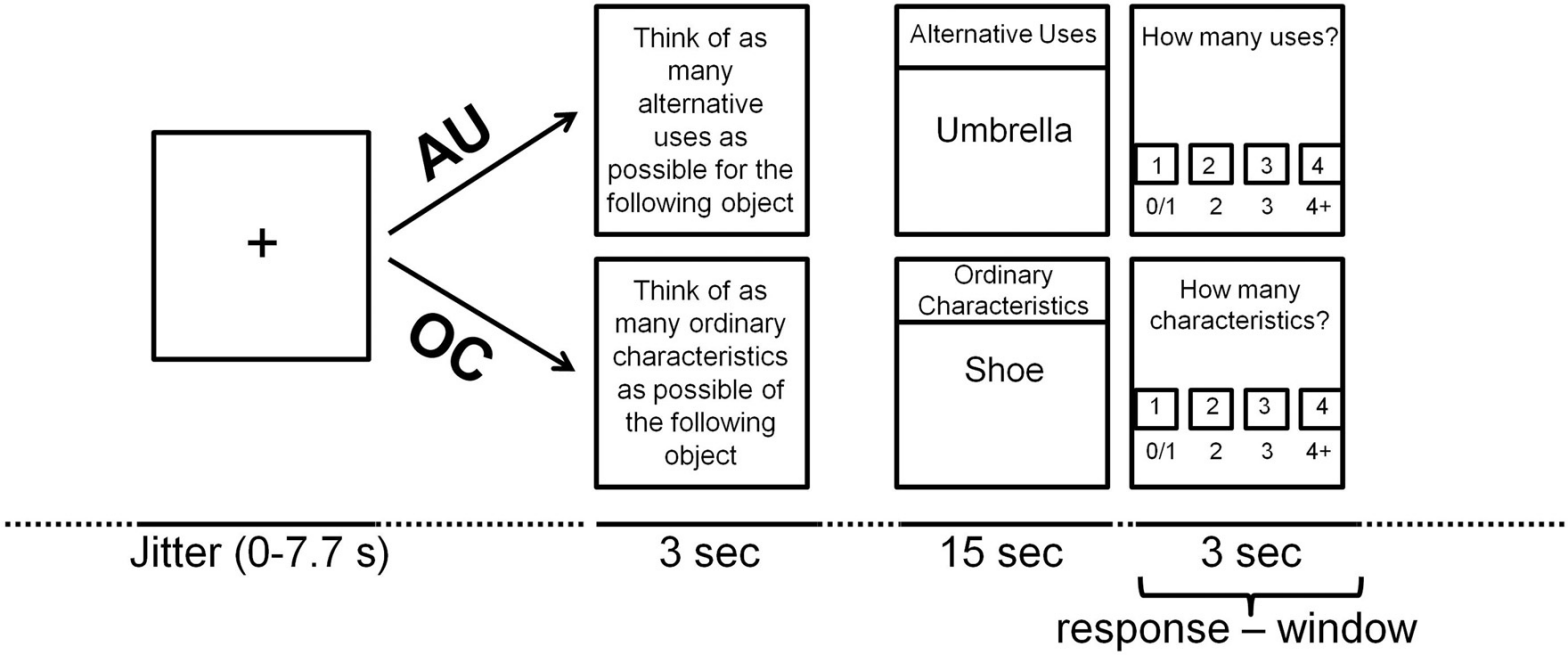
**Time-line of the AUT-scanner task trial (see text for explanation)**.

To examine the neural correlates of divergent thinking, participants performed an adapted version of the Alternative Uses Test (AUT; Guilford, 1950, 1967) inside the MRI scanner while neural activity was measured. The task consisted of two conditions: the free association-related AU condition and the more verbal ability-related Object Characteristics (OC) control condition, based on Fink et al. (2009). During AU trials participants had to think of as many unusual and original uses of a common object as possible (e.g., “umbrella,” example answer: “baseball bat”). During OC trials participants had to think of as many typical characteristics of a common object as possible (e.g., “shoe,” example answer: “fits on a foot”). Each trial started with a 3 s instruction screen to instruct the participant to think of either AU, or common object characteristics. Then, a written item was presented in the middle of the screen for 15 s with the text “OC” or “AU” on the top of the screen during OC and AU trials, respectively, to remind participants of the instruction (see Figure 1). Directly after the target screen, an evaluation screen appeared for 3 s. Participants indicated how many solutions they had found by pressing one of four buttons on a left/right button-box that was attached to their left/right leg respectively; the left middle finger for 0 or 1 solution, the left index finger for 2 solutions, the right index finger for 3 solutions and the right middle finger for 4 or more solutions. Each trial was preceded by a fixation cross that was presented for a variable duration (0–7.7 s) to optimize the event-related design. A total of 40 items (20 AU and 20 OC) were presented in a random order, divided across three blocks with duration of approximately 7 min each. Short breaks were introduced between blocks to prevent fatigue.

For both the AU and OC condition we calculated the percentage of trials on ISO for which participants indicated that they thought of zero or one solution, two solutions, three solutions, and four or more solutions. In addition a composite score was calculated for each condition (AU-score and OC-score). The composite score was the sum of (a) the proportion of zero or one solution times one, (b) the proportion of two solutions times two, (c) the proportion of three solutions times three, and (d) the proportion of four or more solutions times four.

#### Alternative uses test-brick task (AUT-brick)

A computerized version of the AUT was administered outside the scanner to test for convergent validity. This task measures divergent thinking in the verbal domain, similar to the task administered during the scan-session, but now for an extended period of time. Participants were given the name of an object and asked to generate as many AU for the object as possible. In the current version, participants were instructed to generate AU for a *brick* (e.g., Friedman and Förster, 2001). Solutions can be unusual but must be appropriate. Participants were instructed to type their solutions one at the time on a laptop. Answers could be typed for a fixed length of 4 min. *Fluency* scores were computed by counting the number of correct solutions provided. *Flexibility* was measured by the number of solution-categories. An independent trained rater assigned each solution to one of 35 predefined solution-categories (e.g., building aspect; load; toy; Rietzschel et al., 2006; De Dreu et al., 2008). The number of applied solution-categories was counted for each participant individually. O*riginality* was measured on a 5-point scale (from 1 = “not original” to 5 = “highly original”). An independent trained researcher rated the originality of each solution separately according to a previously developed rating scheme to reliably score originality (Rietzschel et al., 2006; De Dreu et al., 2008). Originality scores were calculated for each participant by averaging the rating across all solutions.

#### Verbal fluency test

The verbal fluency test used in the present study was a subtest of the Groninger Intelligentie Test (GIT, Luteijn and van der Ploeg, 1983). The test contained two items: animals and professions, which were applied consecutively. For each item, participants were asked to name as many words as possible that fall within the category of that item, within 1 min. Answers could be given only once. Verbal fluency was scored as the total number of correct answers for both items together.

### Procedure

Outside the scanner, participants received oral instructions and completed a four-trial practice session (2 AU and 2 OC trials) of the scanner task. Then they were acclimated to the MRI environment in a mock scanner. After the scanning phase (during which they performed the scanner task), they completed the WAIS subtests Digit Span and Similarities, the Verbal Fluency test and the 4-min AUT-brick.

### MRI data acquisition

Scanning was performed with a Philips 8-channels SENSE whole-head coil on a 3-Tesla Philips Achieva MRI system (Best, The Netherlands) in the LUMC. Three runs of 167 T2^*^-weighted whole-brain EPIs, preceded by two dummy scans for each run to allow for equilibration of T1 saturation effects, were subsequently acquired (*TR* = 2.2 s; *TE* = 30 ms, flip angle = 80°, 38 transverse slices, 2.75 × 2.75 × 2.75 mm (+10% inter-slice gap). Stimuli were presented running E-prime software (version 1.2, Psychology Tools Inc.) and projected onto a screen at the head of the scanner bore. Participants viewed the stimuli by means of a mirror mounted on the head coil assembly. Head motion was restricted by using pillow and foam inserts that surrounded the head. The maximum movement parameters were below 1.75 mm and the maximum rotation was below 0.5° for all participants and all scans. All anatomical scans were reviewed and cleared by a radiologist.

### MRI data analysis

SPM5 software (www.fil.ion.ucl.ac.uk) was used for image preprocessing and analyses. Images were corrected for slice-time differences, followed by rigid body motion correction. Functional volumes were spatially normalized to EPI templates based on MNI305 stereotaxic space (Cocosco et al., 1997) using a 12-parameter affine transformation together with a non-linear transformation involving cosine basis functions. Data were resampled to 3 mm cubic voxels. Functional volumes were smoothed using an 8 mm full-width half-maximum 3D Gaussian kernel. For each participant, the functional time series were modeled by a series of events convolved with a canonical hemodynamic response function (HRF). Trials were modeled separately based on condition (AU or OC), with the time point of presentation as onset and duration of 15 s, and entered in a general linear model along with a basic set of cosine functions to high-pass filter the data, and a covariate for run effects. In addition, the instruction screen preceding the AU and OC trials and the evaluation screen after trials were modeled separately (onset: presentation onset; duration: 0 ms). Another set of analyses was applied to investigate the process of generating *multiple* solutions, a hallmark of divergent thinking. Here, trials were modeled not only based on the condition (AU or OC), but also on the number of solutions (0/1 or 2+) to make it possible to a) contrast trials with *multiple* AU with trials with *multiple* OC (AU2+ > OC2+); and b) contrast trials with multiple AU with trials with only zero or one AU (AU2+ > AU0/1). The least square parameter estimates of height of best fitting canonical HRF for each condition were used in pair wise contrasts (OC > fixation; AU > fixation; AU > OC; AU2+ > OC2+; AU2+ > AU0/1). The resulting first level contrast images, computed on a subject-by-subject basis, were submitted to group analyses. At the group level, contrasts between conditions were computed by performing one-tailed *t*-tests on these contrasts, treating participants as a random effect, and two-sample *t*-tests to compare age groups. Whole brain fMRI analyses were FDR corrected for multiple comparisons at *p* < 0.05 (voxel level) (Genovese et al., 2002) with at least 10 contiguous voxels. We further conducted whole-brain regression analyses to test for brain behavior relations using the composite AU-score of the scanner task. For whole-brain regression analyses none of the regions survived FDR correction. In addition, we applied the threshold of *p* < 0.001 uncorrected with at least 10 contiguous voxels to overcome the relatively low power inherent to analyses of individual differences, and focused specifically on prefrontal regions in accordance with our hypotheses. Results are reported in the MNI305 stereotaxic space. Brain regions are derived from the SPM anatomy toolbox v1.8 (Eickhoff et al., 2005, 2006, 2007).

### Region-of-interest (ROI) analyses

Region-of-interest (ROI) analyses were performed with MarsBaR toolbox in SPM5 (Brett et al., 2002) to illustrate (1) the activation patterns for the AU and OC conditions within temporo-parietal and prefrontal brain regions and, (2) the correlation between activations related to creative idea generation (AU–OC) and AU performance. ROIs were derived from the whole brain contrasts. The output “contrast estimates” was used. Contrast estimates were derived for each condition relative to baseline (i.e., OC-baseline, AU-baseline). Masked ROIs, including SMG, middle temporal gyrus (MTG) and AG were derived from the contrast AU > OC and were masked with anatomical ROIs derived from the MarsBaR anatomical toolbox.

## Results

### Performance

#### AUT-scanner

To test for creative idea generation performance we conducted a 2 (condition) × 4 (number of solutions) × 2 (age group) mixed-model ANOVA with age group as between-subjects factor. The dependent variable was the number of trials on ISO for which a certain number of solutions was generated. We applied Greenhouse-Geisser corrections if sphericity was violated.

**Figure 2:**
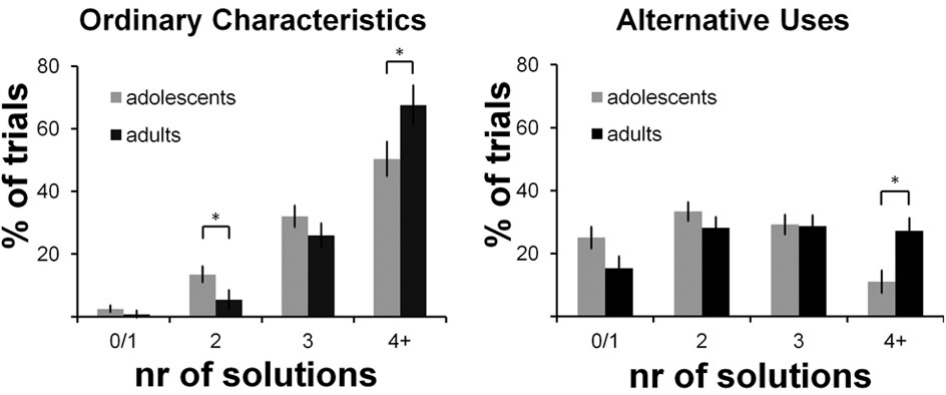
**Performance on the AUT-scanner task**. Percentage of trials (y-axis) for which a certain number of solutions (x-axis) is generated. Results for the Ordinary Characteristics condition are presented on the left; results for the Alternative Uses condition are presented on the right. ^*^ ≤ 0.05.

Results are presented in Figure 2 and show two significant effects: an interaction effect of condition × number with more solutions generated in the OC condition compared to the AU condition [*F*_(3, 123)_ = 58.81, *p* < 0.001; η^2^ = 0.56], and an interaction effect of number × age group [*F*_(3, 123)_ = 5.44, *p* = 0.009; η^2^ = 0.12] with more answers generated by adults than by adolescents. There was no significant condition x number x age group interaction effect [*F*_(3, 123)_ = 0.64, *p* = 0.56, η^2^ = 0.02], indicating that adults were not specifically more creative, but generated more answers in general. *Post hoc* analyses for the number of solutions separately (OC0/1, OC2, OC3, OC4+, AU0/1, AU2, AU3, and AU4+) showed that in both conditions, adults generated four or more solutions more often than adolescents (OC4+: *t* = 3.44, *p* = 0.047; AU4+: *t* = 2.66, *p* = 0.014, corrected for unequal variances); adolescents generated two solutions more often than adults, specifically in the OC condition (*t* = 1.6, *p* = 0.034, corrected for unequal variances). Similar results were obtained for the composite AU- and OC-scores, which are presented in Table 1 [condition effect: *F*_(1, 41)_ = 160.84, *p* < 0.001, η^2^ = 0.80; age group effect: *F*_(1, 41)_ = 7.19, *p* = 0.011, η^2^ = 0.15; condition × age group n.s.: *F*_(1, 41)_ = 0.68, *p* = 0.41, η^2^ = 0.02].

**Table 1:** **Age group performances**.

	**Adolescents**			**Adults**		
	***N***	**Mean**	***SD***	***N***	**Mean**	***SD***
**AUT-SCANNER**						
AU-score^*^	24	2.26	0.50	19	2.68	0.61
OC-score^**^	24	3.32	0.48	19	3.61	0.37
VERBAL FLUENCY^*^	24	41.50	7.30	18	47.56	6.48
**AUT-BRICK**						
Fluency^*^	22	8.45	3.51	17	12.71	5.82
Flexibility	22	6.32	2.36	17	7.82	2.74
Originality	22	1.72	0.27	17	1.61	0.37

AU, alternative uses; OC, ordinary characteristics; AUT, alternative uses test.

* p ≤ 0.05

** p ≤ 0.01.

To test for possible gender effects, we conducted additional repeated measures ANOVA on the scanner task composite scores. No main effect of gender was observed (*p* > 0.05). The interaction effect between condition and gender appeared significant with larger discrepancy between AU-score and OC-score for females relative to males [*F*_(1, 41)_ = 6.25, *p* = 0.02, η^2^ = 0.13]. *Post hoc* analyses showed no significant gender differences for the two measures (AU-score and OC-score) separately (*p's* > 0.05). Interactions with age group (gender × age group, gender × condition × age group) were not significant (*p's* > 0.05).

### Additional tasks

Performances for the tasks taken outside the scanner, the verbal fluency test and the AUT-Brick task, are presented in Table 1.

#### Verbal fluency test

To test for age group differences on verbal fluency an independent samples *t*-test was applied on verbal fluency scores with age group as independent variable. Results revealed a significant age group effect, showing that adults performed better than adolescents: *t*_(40)_ = 2.79, *p* = 0.008. No gender effect or age group x gender effect was observed (*p's* > 0.1).

#### AUT-brick

To examine age group differences for divergent thinking, a multivariate analysis of variances (MANOVA) was performed on the AUT-brick measures fluency, flexibility, and originality. Results showed a significant age group effect [*F*_(4, 34)_ = 3.34, *p* = 0.02]. *Post hoc* analyses showed that the effect was driven by a significant age group effect for AUT-brick fluency with better performance for adults compared to adolescents [AUT-brick fluency: *t*_(37)_ = 2.66, *p* = 0.01, corrected for variance differences]. Age group differences were only marginally significant for AUT-brick flexibility [*t*_(37)_ = 1.84, *p* = 0.07]. No age group effects were observed for AUT-brick originality. Additional analyses on gender and gender × age group effects revealed no significant results. On a behavioral level, these results are in line with performance on the scanner task, indicating that adults showed greater fluency in general.

### Correlations

To validate the processes that are involved in the AU and OC conditions, bivariate correlations were estimated between AU- and OC-scores of the scanner task, and performances on the verbal fluency test and AUT-brick task. Results are presented in Table 2. Significant correlations were observed for OC-score with both fluency measures; verbal fluency (*r* = 0.44, *p* < 0.001) and AUT-brick fluency (*r* = 0.36, *p* = 0.02), but not for OC-score with AUT-brick flexibility, or AUT-brick originality. AU-score correlated significantly with AUT-brick fluency (*r* = 0.54, *p* < 0.001) and AUT-brick flexibility (*r* = 0.36, *p* = 0.02), and marginally with AUT-brick originality (*r* = 0.30, *p* = 0.07), but not with verbal fluency (*r* = 0.26, *p* > 0.1). Similar results were obtained when analyses were controlled for gender. When controlling for age, results showed some deviations: OC-scores correlated no longer with AUT-brick fluency scores (*r* = 0.26, *p* = 0.12); AU-scores correlated only marginally with AUT-brick flexibility scores (*r* = 0.29, *p* = 0.08), but significantly with AUT-brick originality scores (*r* = 0.40, *p* = 0.01). In all, these results support the differentiation between the two conditions with the AU condition related to creativity-related divergent thinking aspects and OC condition associated with more verbal ability-related fluency capacities (Fink et al., 2009).

**Table 2:** **Bivariate correlations for fluency and creativity measures**.

	**AUT-scanner**	
	**AU-score**	**OC-score**
Verbal fluency	0.26	0.44^**^
AUT-brick fluency	0.54^**^	0.36^*^
AUT-brick flexibility	0.36^*^	0.15
AUT-brick originality	0.30^~^	0.07

AU, alternative uses; OC, ordinary characteristics; AUT, alternative uses test.

* p ≤ 0.05

** p ≤ 0.01

~ p ≤ 0.10.

### fMRI results

**Figure 3:**
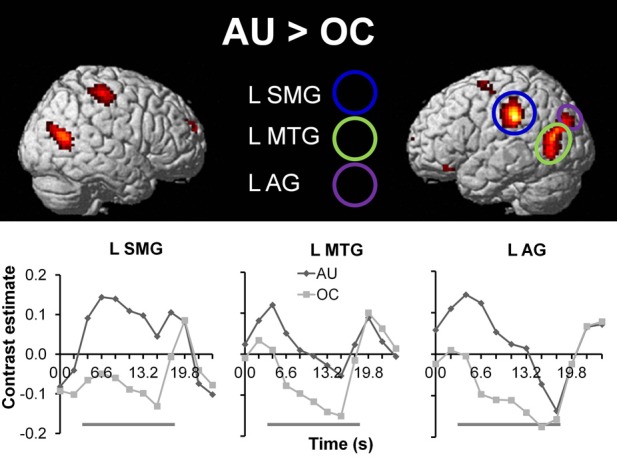
**Top: Whole brain results for the contrast AU > OC (*N* = 43; *p* < 0.05, FDR corrected (voxel level), >10 contiguous voxels)**. Below: Time series for the anatomically masked functional ROIs for the AU and OC conditions, with onset of instruction screen at time = 0 s. The gray beams beneath the graphs represent object presentation (time = 3.0–18.0 s) during which participants are required to generate solutions. Time series are presented for illustrative purposes only. AU, Alternative Uses; OC, Ordinary Characteristics; L SMG, left supramarginal gyrus; L MTG, left middle temporal gyrus; L AG, left angular gyrus.

To extract the neural correlates of creative idea generation we conducted whole-brain voxel-wise *t*-tests on activation levels for the contrast AU > OC across all participants (*N* = 43). Results revealed a number of regions including left SMG, left and right MTG, and left AG (FDR corrected, *p* < 0.05; see Figure 3 and Table 3), which is in line with prior studies (Fink et al., 2009, 2010). The opposite contrast OC > AU showed increased activity for retrieval of characteristics relative to AU for common objects in a number of different brain regions (see Table 4). Results were most pronounced in left and right posterior SMG/anterior AG and thereby resemble previous findings by Fink et al. (2010).

**Table 3:** **Neural activations for the contrast AU > OC**.

**Brain regions**	**L/R**	***K***	***Z*-value**	**MNI coordinates**		
			**Peak voxel**	***x***	***y***	***z***
Supra marginal gyrus	L	189	6.63	−60	−30	36
Inferior parietal cortex (PGp), middle temporal Gyrus	L	265	5.01	−42	−84	30
			4.25	−54	−66	0
			4.04	−45	−63	9
Anterior cingulate cortex, middle orbital gyrus	L	834	4.91	−3	48	0
			4.86	−9	48	−6
			4.76	0	51	12
Postcentral gyrus	R	199	4.73	36	−30	51
Middle temporal gyrus	R	160	4.49	48	−63	15
			3.81	51	−75	21
Hippocampus	L	26	4.06	−24	−12	−18
Superior frontal gyrus (BA6)	L	55	3.99	−21	−6	60
Calcarine gyrus	L	65	3.79	−6	−51	6
			3.47	−9	−60	9
Inferior frontal gyrus (p. Orbitalis)	L	31	3.61	−30	33	−15
Inferior frontal gyrus (p. Opercularis)	L	12	3.44	−51	6	24

Abbreviations: MNI, montreal neurological institute; L, left hemisphere; R, right hemisphere.

Significance threshold: p < 0.05, FDR corrected (voxel level); >10 voxels.

**Table 4:** **Neural activations for the contrast OC > AU**.

**Brain regions**	**L/R**	***K***	***Z*-value**	**MNI coordinates**		
			**Peak voxel**	***x***	***y***	***z***
Angular gyrus (hIP3), inferior parietal lobule (PFm), supra marginal gyrus (hIP1)	R	2745	7.54	33	−63	48
			5.99	51	−51	48
			5.11	48	−63	48
Middle temporal gyrus, precentral gyrus (area 4a, 3b), Angular Gyrus (hIP1)	L	6441	6.87	−66	−51	48
			6.14	−36	−42	39
			6.02	−33	−36	−6
Inferior temporal gyrus, rolandic operculum (OP 4)	R	1069	5.43	57	−24	57
			4.35	66	−63	42
			4.21	63	−39	−18
Inferior occipital gyrus (hOC3v (V3v), fusiform gyrus, middle occipital gyrus	L	1025	4.91	−27	−27	−18
			4.78	−33	−3	12
			4.55	−39	−93	−9
Middle cingulate cortex	R	221	4.69	3	−72	−15
Precentral gyrus (area 6)	R	61	3.62	21	−87	−3
Middle temporal gyrus	L	35	3.60	−54	−33	33
Medial temportal pole	R	12	2.71	48	−27	60
Lingual gyrus (area 17)	R	19	2.54	9	6	−24
			2.50	3	9	−24

Abbreviations: MNI, montreal neurological institute; L, left hemisphere; R, right hemisphere.

Significance threshold: p < 0.05, FDR corrected (voxel level); >10 voxels.

We conducted a second set of analyses to examine neural correlates for trials on which multiple solutions were generated, a crucial characteristic of divergent thinking success. Contrasting trials on ISO for which participants indicated that they had found two or more AU with trials on ISO for which they had thought of two or more OC (AU2+ > OC2+), revealed similar results as the contrast AU > OC, including left SMG, left AG and bilateral MTG. However, additional activation was observed in several regions, including left middle frontal gyrus (MFG) and left inferior frontal gyrus (IFG) pars Triangularis (see Table 5). A direct comparison of AU trials with two or more solutions and trials with zero or one solution (AU2+ > AU0/1) revealed no significant effects. However, applying a more liberal significance threshold (*p* < 0.001, uncorrected) revealed activations mainly in the left hemisphere, including left MFG and left IFG pars Triangularis (see Table 6). These findings are in congruence with the findings for contrast AU2+ > OC 2+ and indicate that the differences between the contrasts AU > OC and AU2+ > OC2+ are not the result of differences between OC0/1 and OC2+ trials.

**Table 5:** **Neural activations for the contrast AU 2+ > OC2+**.

**Brain regions**	**L/R**	***K***	***Z*-value**	**MNI coordinates**		
			**Peak voxel**			
Inferior parietal cortex (PGp), middle temporal gyrus	L	106	5.34	−42	−84	30
			3.72	−42	−63	15
			3.37	−51	−69	15
Fusiform gyrus	L	40	4.39	−24	−33	−21
			3.43	−33	−42	−21
Hippocampus	L	18	4.37	−24	−15	−18
Postcentral gyrus (area 2)	R	39	4.34	36	−33	48
Anterior cingulate cortex, middle orbital gyrus	L	147	4.34	0	36	−6
			4.27	−9	51	−6
			3.64	−3	48	0
Olfactory cortex	L	27	4.24	0	12	−6
Inferior frontal gyrus (p. Opercularis; area 44), rolandic operculum	L	36	4.14	−51	6	24
			3.36	−42	−3	18
Middle frontal gyrus (Area 6)	L	62	4.13	−24	0	60
			3.42	−24	−9	48
Middle temporal gyrus/middle occipital gyrus (PGp)	R	65	4.07	48	−63	15
			3.64	51	−75	24
Middle temporal gyrus	L	28	4.05	−54	−66	−3
Inferior frontal gyrus (p. Orbitalis)	L	29	4.03	−27	33	−15
Superior frontal gyrus	L	11	3.88	−18	21	39
Inferior parietal lobule (area 2/hIP3)	L	22	3.65	−39	−39	48
			3.25	−30	−36	39
Superior frontal gyrus	L/R	17	3.61	3	57	12
Inferior frontal gyrus (p. Triangularis), middle frontal gyrus	L	24	3.60	−45	33	12
			3.54	−45	39	18
Calcarine gyrus	L	12	3.45	−9	−48	6

Abbreviations: MNI, montreal neurological institute; L, left hemisphere; R, right hemisphere.

Significance threshold: p < 0.05, FDR corrected; >10 voxels.

**Table 6:** **Neural activations for the contrast AU 2+ > AU 0/1**.

**Brain regions**	**L/R**	***K***	***Z*-value**	**MNI coordinates**		
			**Peak voxel**			
Anterior cingulate cortex, cingulate gyrus	L/R	33	4.40	−6	9	24
			3.24	3	0	27
Rolandic operculum, precentral gyrus (area 44)	L	71	4.35	−48	3	12
			4.17	−48	3	21
			3.43	−39	−3	15
Postcentral gyrus (area 1), inferior parietal lobule (area 2/hIP3), postcentral gyrus (area 2/3b)	L	122	3.91	−42	−30	63
			3.84	−36	−39	48
			3.55	−42	−27	48
Precentral gyrus (area 6)	L	24	3.84	−24	−9	66
			3.27	−30	−21	63
Cerebellum (lobule VI, hem)	R	14	3.77	27	−51	−30
Superior frontal gyrus	L	25	3.76	−15	21	54
			3.58	−15	12	57
Inferior frontal gyrus (p. triangularis)	L	53	3.63	−45	33	15

Abbreviations: MNI, Montreal Neurological Institute; L, Left hemisphere; R, Right hemisphere.

Significance threshold: p < 0.001, uncorrected (voxel level).

To test for developmental differences, whole-brain two-sample *t*-tests (adolescents vs. adults) were conducted on the contrast AU > OC. There were no age group differences (significance threshold: *p* < 0.05 FDR corrected). There were also no age group differences when we analyzed only those trials on ISO for which participants gave at least two solutions (AU2+ > OC2+).

### Individual differences

**Figure 4:**
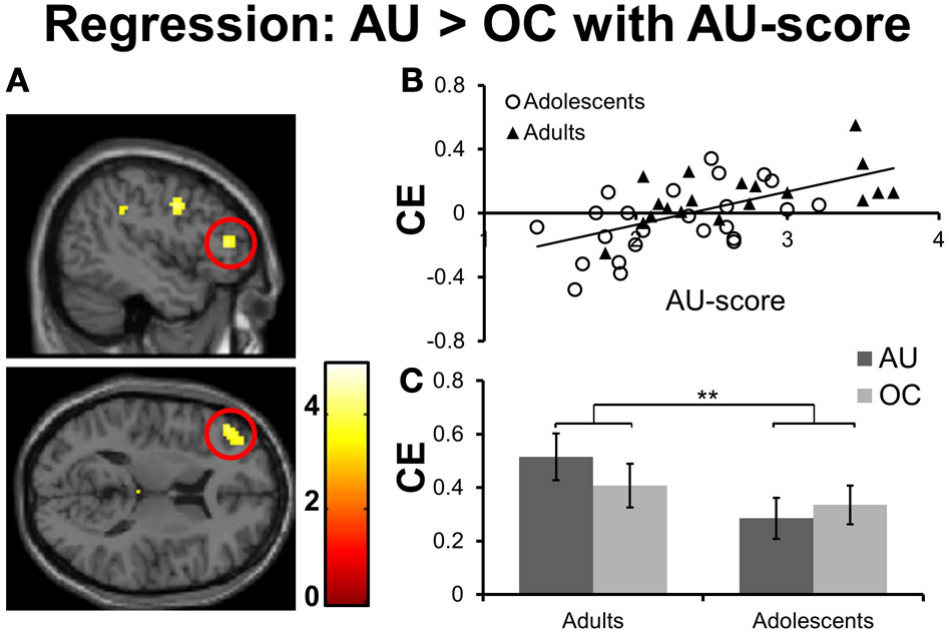
**(A)** Whole brain results for the regression of AU-score on the contrast AU > OC, thresholded at *p* < 0.001, uncorrected; >10 contiguous voxels (section coordinates: *X* = −45, *Z* = 12). Color–scale represents *t*-values. **(B)** Correlation between creative thinking related activation (AU-OC) for the left IFG/MFG (ROI-peak-value at MNI coordinates −45, 39, 12) and AU-score for adolescents (open circles) and adults (filled triangles). **(C)** Contrast estimates for AU and OC conditions relative to baseline for left IFG/MFG. Results for adolescents are presented left, results for adults are presented right. CE, Contrast estimate. ^**^p ≤ 0.01.

To test for brain regions directly related to divergent thinking performance (fluency), we conducted whole-brain voxel-wise regression analyses on the contrast AU > OC with performance on AU trials (AU- score of the scanner task, see methods section) as covariate of interest. No significant findings were observed at the threshold *p* < 0.05 FDR corrected. However, when the threshold was lowered to *p* < 0.001, uncorrected, >10 contiguous voxels, significant correlations were found in a number of regions for which we had a priori hypotheses (see Table 7). Specifically, activation levels for the contrast AU > OC were correlated with creative performances in a region in the left lateral PFC (MFG and IFG pars Triangularis, see Figures 4A,B). Notably, this region overlapped with the left MFG/IFG regions that were significantly more activated for AU2+ trials relative to AU0/1 trials. As such, our results show both within- and between-subject support for a significant role for left lateral PFC in divergent thinking. In addition to left lateralized brain regions, the regression analyses with AU-scores (*p* < 0.001, uncorrected for multiple comparisons) revealed significant results in the right hemisphere, including right MFG and right IFG pars Triangularis (see Table 7). Thus, while successful divergent thinking (2+ solutions; within subjects) was associated with increased *left* lateralized prefrontal activations, divergent thinking performance (AU- score; between subjects) related to activity changes in both left and right prefrontal cortices.

**Table 7:** **Neural activations for the regression AU > OC with AU-scores**.

**Brain regions**	**L/R**	***K***	***Z*-value**	**MNI coordinates**		
			**Peak voxel**			
Supplementary motor area (bilateral), superior medial gyrus (L)	L/R	93	4.44	9	15	48
			3.48	−3	18	45
			3.43	−9	27	36
Cerebellum (lobule VI hem, vermis)	R	190	4.24	15	−75	−24
			4.13	30	−63	−27
			3.54	6	−66	−24
Middle frontal gyrus	R	51	4.00	30	9	51
			3.81	27	−3	45
Middle frontal gyrus	R	48	3.96	42	42	21
Precentral gyrus, superior frontal gyrus (area 6)	L	30	3.90	−33	−9	66
			3.54	−27	−3	69
			3.38	−3	−27	21
Precentral gyrus	L	63	3.85	−36	0	33
			3.82	−45	0	39
Inferior frontal gyrus (p. triangularis), middle frontal gyrus	L	45	3.76	−45	39	12
			3.69	−36	48	15
			3.31	−33	51	24
Cerebellum (lobule VI, hem)	L	23	3.75	−9	−78	−21
Inferior Frontal gyrus (p. orbitalis)	R	43	3.76	42	27	−9
Inferior parietal lobule (hIP1)	L	22	3.51	−39	−45	33
			3.13	−42	−51	42
Supplementary motor area (area 6)	L	10	3.26	0	0	57

Abbreviations: MNI, montreal neurological institute; L, left hemisphere; R, right hemisphere.

Significance threshold: p < 0.001, uncorrected; >10 voxels.

We hypothesized that adolescents would show immature divergent thinking performance related to immature PFC activation patterns. To test these hypotheses we performed ROI analyses on the contrast estimates of the left IFG/MFG cluster, which were derived from the regression analyses described above. First, we examined whether age group differences were present using a 2 (condition) × 2 (age groups) mixed ANOVA on contrast estimates for AU and OC conditions relative to baseline (AU- baseline; OC- baseline). Results revealed a significant age group × condition interaction effect [*F*_(1, 41)_ = 7.21, *p* = 0.01, η^2^ = 0.15; see Figure 4C). Adults showed larger condition effects than adolescents, which is in line with our hypothesis that adolescents do not yet recruit left lateral PFC for creative thinking at a similar level as adults.

Second, we applied a 2 (conditions) × 2 (age groups) mixed ANOVA with AU-scores entered as covariate, given that age groups differed in performance. Results revealed a significant interaction effect for condition × AU-score [*F*_(1, 41)_ = 15.56, *p* < 0.001, η^2^ = 0.28] but no significant age group x condition or age-group × condition × AU-score interaction effect (*p*'s > 0.10). These results indicate that divergent thinking performance predicts left IFG/MFG activation for creative idea generation across both age groups, and adolescents do not recruit these relevant brain regions at an adult level yet.

### Gender differences

To test for possible gender differences (see Abraham et al., 2012) we applied explorative whole-brain analyses. No significant effects were observed (significance threshold: *p* < 0.05, FDR corrected, >10 contiguous voxels). Additional mixed ANOVA's on contrast estimates for the AU and OC conditions relative to baseline for the four functional ROIs (lMTG, lSMG, lAG, L IFG/MFG) showed no effects for lAG and lIFG/MFG. For lMTG and lSMG, significant interaction effects were observed for gender × condition (AU > OC) with larger discrepancies for males relative to females [SMG: *F*_(1, 41)_ = 4.88, *p* = 0.03, η^2^ = 0.10; MTG: *F*_(1, 41)_ = 6.59, *p* = 0.01, η^2^ = 0.14].

## Discussion

The aim of the study was to better understand distinctive neural activation patterns supporting creative idea generation in adolescents and adults. For this purpose, we applied an adapted version of the AUT (Guilford, 1950, 1967; Fink et al., 2009, 2010) while scanning with fMRI. Significant correlations with tests performed outside the scanner (AUT-brick task and verbal fluency test) validated the two conditions used to extract processes underlying creative idea generation (AU and OC). On the behavioral level, adults outperformed adolescents on generating AU as well as naming OC. The fMRI data yielded three important findings: (1) consistent with prior studies, we found increased activation of left SMG and AG, as well as left MTG and medial frontal cortex for creative idea generation relative to naming OC; (2) trials during which participants generated multiple solutions (AU2+ > OC2+ and AU2+ > AU0/1), a hallmark of divergent thinking, revealed additional left IFG/MFG activation; (3) individual differences analyses showed that performance on the AU trials predicted left IFG/MFG activations related to creative idea generation, and adults recruited this brain region more than adolescents. The discussion is organized along the lines of these three main findings.

### Neural correlates of creative idea generation

Consistent with prior studies (e.g., Starchenko et al., 2003; Bechtevera et al., 2004; Fink et al., 2009, 2010), generating AU relative to naming OC resulted in increased activity in mainly left hemisphere regions, including AG, SMG, and MTG. These temporo-parietal regions are argued to be critically involved in verbal creative thinking (Bechtevera et al., 2004; Fink et al., 2009). One possible role of these brain regions in the current task concerns semantic processing: activation of an object's semantic information is likely a precursor for generating possible uses of that object. Several prior studies have demonstrated that the AG, SMG (posterior), and MTG are involved during semantic tasks (e.g., Jung-Beeman, 2005; Vigneau et al., 2006; Binder et al., 2009). Moreover, MTG and SMG have been specifically related to tool use and action knowledge, including semantic information of tools and imaginative tool use (Beauchamp et al., 2002; Johnson-Frey, 2004; Lewis, 2006). It is likely that processing these types of information on tool use is especially profitable when thinking about AU of objects. Furthermore, activation of the SMG and AG may contribute to the flexible character of creative thinking: prior neuroimaging and clinical studies indicate the importance of these temporo-parietal regions in the flexible switching between tasks (e.g., Sohn et al., 2000; see also Starchenko et al., 2003; Bechtevera et al., 2004) and between attention foci (e.g., Humphreys et al., 1994).

Besides the anticipated temporo-parietal regions, creative idea generation was associated with activation in e.g., a large cluster within the medial PFC, including (anterior) cingulate cortex (ACC). One possible interpretation for these results is that creative idea generation involves monitoring of information retrieval. The ACC is commonly associated with error or conflict monitoring processes (e.g., Botvinick et al., 2004). This interpretation would be in congruence with the idea that flexible processing requires an evaluation mechanism (“idea monitor”) to judge the appropriateness of generated responses (Dietrich, 2004; Iyer et al., 2009; Nijstad et al., 2010).

Some regions that were more active when thinking about AU than OC, such as the AG and the medial PFC, are part of the default mode network (Raichle et al., 2001). These areas have previously been associated with free thinking, mentalizing and mind wandering (e.g., Mason et al., 2007; Buckner et al., 2008; Gruberger et al., 2011; Christoff, 2012). It is reasonable to assume that these processes are also involved when thinking “out of the box.” Indeed, mind wandering has been thought of as micro *incubation* (Sawyer, 2011), a process during which one refrains from conscious thought and after which a creative insight “suddenly” appears in the conscious mind (see e.g., Dijksterhuis and Meurs, 2006). An interesting question for future research is to examine activations of resting state networks in combination with divergent thinking tasks to better understand the role of these networks in divergent thinking (see also Takeuchi et al., 2011a,b).

### Performance-related activation of lateral prefrontal cortex

The results in this study showed that generating multiple creative ideas (within subjects) and better creative performance (between subjects) were both related to increased activity in left lateral PFC. This brain region is generally associated with cognitive control functioning and has been shown to be involved in switching between semantic (sub)categories (Hirshorn and Thompson-Schill, 2006). These findings are in line with the conceptions that creative thinking involves cognitive flexibility and working memory (e.g., Vartanian, 2009; Zabelina and Robinson, 2010; De Dreu et al., 2012), as it refers to the generation of original and useful ideas by combining already stored information (Dietrich, 2004). Furthermore, the brain-behavior correlations support the hypothesis that PFC activity is predictive of divergent thinking performance, whereas temporo-parietal activations are related to creative thinking in general. Therewith, our results complement previous studies showing activation levels for prefrontal brain regions discriminative concerning divergent thinking (e.g., Carlsson et al., 2000; Chavez et al., 2004; Chavez-Eakle et al., 2007; Gibson et al., 2009). Creating AU relative to OC of objects revealed activations in *left* lateral PFC, whereas better, relative to poorer, performance on the AU task was associated with larger lateral PFC activations in *both* hemispheres. These findings indicate that divergent thinking in general is dominated by left hemisphere activation, but creativity performance is related to the level of recruitment of both hemispheres. It should be noted that performance-related activation was also found in other areas for which we did not have a priori hypotheses. Future studies should examine the robustness of the role of PFC in divergent thinking and the possible role of other areas and their connections with PFC.

### Developmental differences

One additional question that was addressed in this study was whether there would be a difference in neural recruitment during divergent thinking in adolescents compared to adults. This hypothesis was based on the assumption that there is continued development of brain regions implicated in divergent thinking, especially of the lateral PFC (Shaw et al., 2008; Giedd and Rapoport, 2010; Luna et al., 2010), although the specifics are currently debated, with some studies reporting more activation in adolescents compared to adults, and others reporting less activation in lateral PFC in adolescents compared to adults (see Crone and Dahl, 2012 for an overview). Behaviorally, our results indicate that adolescents are still developing creative abilities, showing immature fluency measures, but not originality or flexibility measures. Notably, these results deviate from a prior study, showing immature originality, but not fluency performance (Kleibeuker et al., 2013). A possible explanation for this difference is that the adolescent age groups slightly differed in mean age, with older participants in the current study. Another possible explanation concerns a shift in the balance of quality (originality) and quantity (fluency) of answers whereby participants in the current study focused more on quality rather than quantity of answers, as a result of the emphasis on *alternative* object uses in the scanner task.

The current neuroimaging findings revealed no age differences at the whole brain level. However, region-of-interest analyses revealed that the left lateral PFC, which was related to individual differences in divergent thinking performance, was more activated in adults than in adolescents. One possible interpretation is that adolescents were not yet able to recruit these task-relevant brain regions to a mature level for the task at hand. Indeed, creativity promoting complex abilities of controlling thought processes and flexibly changing perspectives are thought to develop throughout adolescence (Wu and Chiou, 2008). Specifically, prior studies have suggested that attentional inhibition and cognitive flexibility are still developing in adolescence (Huizinga et al., 2006). However, it should be noted that this PFC region was extracted from an individual differences contrast and age-effects disappeared when performance was entered as a covariate. Moreover, there were no general age effects for the main contrast AU > OC. A possible interpretation is that higher fluency/divergent thinking performance is associated with higher IFG/MFG activation, and that adults may achieve higher performance by stronger recruitment of this region. How exactly inhibition and cognitive flexibility, and their developmental trajectories, relate to creative cognition, remains an important question for future studies. To our knowledge, this is the first study comparing adults' and adolescents' brain activity during creative divergent thinking, and future studies will provide more insight into these compelling questions.

### Gender differences

Although males and females did not differ in task performance for the two scanner task conditions (AU and OC) separately, there was a small gender difference in relative performance for these two conditions with females showing larger discrepancies between retrieval success and creative thinking. These results might be the consequence of slight differences in creative thinking strategies (McCarthy et al., 2012). Accordingly, behavioral effects were accompanied with larger creative thinking related activations of left MTG and SMG in males relative to females. These results are in line with previous findings in research on gender differences in creative thinking (Abraham et al., 2013). The lack of age related differences in these gender effects indicate that possible discrepancies in creative thinking strategies are already present in adolescence.

### Limitations and future directions

Some limitations of the current study should be taken into account when drawing conclusions on the underlying processes of creative thinking. First of all, one should be cautious with generalizing the present results. The current study examined neural correlates of flexible, divergent thinking as one of the key drivers underlying creative performance and original ideation. However, the DPCM (Baas et al., 2008; De Dreu et al., 2008; Nijstad et al., 2010) highlighted another pathway, namely perseverance. Future research is needed to develop fMRI tasks that capture perseverance, so that the neural correlates of flexible processing as well as perseverance can be examined alone and in combination. Here we applied an adapted version of the AUT where creative thinking was operationalized by the processes underlying idea generation. These processes likely involve e.g., semantic processing and (verbal) working memory, inherent to the identity of the task. In the present fMRI design creative *performance* was operationalized as the number of generated AU for common objects as indicated by the participant, implying an indirect measure of creative performance. Another limitation of this study, regarding the interpretability of developmental changes, is that the results were cross-sectional and not longitudinal. The reliability of the observed age differences is therefore limited. Future studies are necessary to understand the development of processes that underlie creative idea generation. Moreover, for future research it would be interesting to focus on possible improvements and related changes in brain recruitment in adolescents, applying practice/training of idea generation. These results are expected to give better understanding of the possibilities and limitations of the adolescent brain regarding creative idea generation.

## Conclusion

Taken together, the results of this study demonstrated that creative idea generation in general involves recruitment of mainly left lateralized parietal and temporal brain regions that are associated with semantic activation, imagination and tool use, including AG, SMG, and MTG. However, generating *multiple* creative ideas, a hallmark of divergent thinking, shows additional lateral PFC activation, which is not yet optimized in middle adolescence.

### Conflict of interest statement

The authors declare that the research was conducted in the absence of any commercial or financial relationships that could be construed as a potential conflict of interest.
